# Beliefs about the effects of health behaviors on cancer risk: findings from the 2022 Health Information National Trends Survey

**DOI:** 10.1007/s10552-025-02051-x

**Published:** 2025-08-20

**Authors:** Christine M. Swoboda, Olivia Pardi, Ann Scheck McAlearney

**Affiliations:** 1 The Center for the Advancement of Team Science, Analytics, and Systems Thinking in Health Services and Implementation Science Research (CATALYST), College of Medicine, The Ohio State University, 700 Ackerman Rd, Suite 4101, Columbus, OH 43202 USA; 2 Department of Family and Community Medicine, College of Medicine, The Ohio State University, Columbus, OH USA

**Keywords:** Cancer beliefs, Cancer prevention, Cancer risk behaviors, Lifestyle behaviors

## Abstract

**Purpose:**

While some health behaviors may help reduce risk of cancer, beliefs about which behaviors affect cancer risk and the preventability of cancer vary within the population. The purpose of this study was to assess beliefs about cancer prevention behaviors and cancer fatalism.

**Methods:**

The sample consisted of 6252 respondents from the 2022 Health Information National Trends Survey (HINTS). Multivariable ordinal regression models were used to find the odds of believing health behaviors increased cancer risk by demographics, health behaviors, and cancer fatalism beliefs.

**Results:**

Female respondents had higher odds of believing drinking alcohol, processed meat, red meat, and fast food increased cancer risk a lot (all *p* < 0.05). Black, Hispanic, and Other race respondents had higher odds of believing that processed meat increases cancer risk, while Hispanic respondents also had higher odds of believing soda, alcohol, red meat, fast food, and not enough fruits and vegetables increase risk (all *p* < 0.05). Those who feel “A lot” or “Some” progress has been made in preventing cancer had higher odds of believing drinking soda (OR (95% CI) 1.49 (1.16–1.91)), drinking alcohol (OR (95% CI) 1.82 (1.28–2.59)), processed meat (OR (95% CI) 1.84 (1.11–3.04)), red meat (OR (95% CI) 1.67 (1.24–2.24)), and not enough fruits and vegetables (OR (95% CI) 1.39 (1.01–1.92)) increased cancer risk.

**Conclusion:**

While most people believe nutrition, alcohol, and sleep have some effect on cancer risk, women, older adults, and races other than White are more likely to believe multiple behaviors increase risk a lot. Cancer prevention information should help inform which behaviors affect risk but also focus on helping people make realistic behavior changes.

## Introduction

The American Cancer Society predicts that over two million new cancer cases will be diagnosed and over 600,000 deaths from cancer will occur in 2024 [1]. Cancer will affect about 39.5% of adults in the United States (U.S.) during their lifetime [2], but between 30 and 50% of cancer cases may be preventable [3]. Behaviors including not smoking, drinking less alcohol, consuming a healthy diet, regular physical activity, stress reduction, and adherence to screening guidelines may decrease cancer risk, but these behaviors vary among the population [4]. Racial and ethnic minorities and those with lower socioeconomic status have reduced access to healthy food, increased exposure to environmental contaminants, higher likelihood of employment-related exposure to carcinogens, and less access to care [5, 6]. Correspondingly, there are disparities in cancer incidence and death that are related to these factors [7].

Fatalistic beliefs about cancer are characterized by pessimism, powerlessness, feelings that cancer is always fatal, feeling cancer is unavoidable, confusion regarding prevention recommendations, and doubts regarding preventability [8, 9]. These beliefs vary by levels of health literacy, education, and income, as well as by race, age, and sex [8–12]. In prior research, those with limited health literacy, lower education, and non-Hispanic Black race more frequently agreed that “there is not much you can do to lower your chances of getting cancer,” and younger, female, and lower education respondents were more likely to agree that “it seems like everything causes cancer” [9]. In another study, low income, low educational attainment, and non-Hispanic Black respondents had more fatalistic beliefs but also lower perceived risk of cancer and fewer worries about cancer [12]. These differences in fatalistic beliefs may pose risks, however, as believing that cancer is unavoidable may impact cancer prevention behaviors [8].

Prior research has found associations between cancer fatalism and diet, exercise, and smoking, as well as cancer screening and health information seeking behaviors [8, 9, 13–16]. Those who held fatalistic beliefs about cancer had a lower likelihood of exercising weekly, were less likely to be a nonsmoker, and were less likely to eat five servings of fruits and vegetables daily [8, 15–17]. Respondents with fatalistic beliefs also had lower rates of screening for cervical, colorectal, breast, and prostate cancer [13, 14]. Cancer fatalism was similarly associated with information avoidance and reduced health information seeking, possibly due to low perceived utility of following recommendations for cancer prevention [9, 18, 19].

Because of the association between cancer fatalism and adherence with cancer prevention and screening behaviors, it is important to know the characteristics of individuals who have these fatalistic beliefs. Additionally, little research has been conducted to improve our understanding about which specific behaviors people believe affect cancer risk. The purpose of this study was to assess beliefs about cancer prevention behaviors and cancer fatalism and to learn more about the link between these beliefs, using data from the 2022 iteration of the Health Information National Trends Survey. We hypothesized that those who believe that diet, alcohol, and sleep affect cancer risk will be less likely to hold fatalistic cancer beliefs.

## Methods

### Study sample

This study uses cross-sectional data from the 2022 iteration of The Health Information National Trends Survey (HINTS). HINTS was created by the National Cancer Institute and asks questions about cancer beliefs, health behaviors, health information use, healthcare beliefs and use, demographic information, and opinions about various health topics. HINTS was fielded 16 times between 2003 and 2022 and is administered to random samples of adults in the United States. The inclusion criteria consist of non-institutionalized adults aged 18 or older residing in the U.S. at the time of the survey. All HINTS administrations are de-identified before being made available and have been approved by the Westat Institutional Review Board and deemed exempt from approval or consent for subsequent analyses [20]. The HINTS survey is sent with a $2 incentive to encourage participant completion [21].

The 2022 administration of HINTS had 6,252 responses and a response rate of 28.1% [21]. Data were collected by mail and online. Mailed surveys were collected in two stages. The first stage randomly selected residential addresses from the U.S. Postal Service and the second stage selected the adult in the household with the nearest upcoming birthday [21]. Data from only 2022 was used in this analysis because the questions about which behaviors affect cancer risk were new in this iteration.

Data were analyzed using survey weighting to make the results more generalizable to the U.S. population. Survey weights were designed based on population estimates from the American Community Survey, and jackknife replicate weights were used to provide bias-corrected variance estimates [22].

### Measures

The dependent variables of interest in this study were assessed using the questions: “How much do you think that … could increase a person’s chance of developing cancer?” with the behaviors “drinking soda or other sugar-sweetened drinks,” “drinking alcohol,” “eating too much processed meat,” “eating too much red meat,” “eating too much fast food,” “not eating enough fruits and vegetables,” and “not getting enough sleep” and the responses “A lot,” “A little,” “Not at all,” and “Don’t know.” For regression analyses, the responses were turned into ordinal variables: Not at all = 0, A little = 1, and A lot = 2 to assess the odds of stronger feelings that each factor increased cancer risk; the “Don’t know” responses were excluded from analysis.

Independent variables assessing other cancer beliefs and health behaviors were used. The questions “How much progress has been made in preventing cancer” and “How much progress has been made in curing cancer” had the responses “A lot,” “Some,” “A little,” “Almost none,” and “Don’t know” which were changed to “A little/Almost none” and “A lot/Some” for analysis. Four variables about cancer fatalism included “It seems like everything causes cancer,” “There’s not much you can do to lower your chances of getting cancer,” “There are so many different recommendations about preventing cancer, it’s hard to know which ones to follow,” and “When I think about cancer, I automatically think about death,” with the options “Strongly agree,” “Somewhat agree,” “Somewhat disagree,” and “Strongly disagree,” which were turned into binary variables “Strongly/Somewhat agree” and “Strongly/Somewhat disagree” for analysis. Behavioral variables included were hours of sleep per night, which was a number from 0 to 24 that was recoded to “5 or less,” “6/7 h,” and “8 or more,”; the question “Think about the last time you ordered food in a fast food or sit down restaurant, did you notice calorie information listed next to the food on the menu or menu board?” with the options “Yes” and “No,”; and number of days per week drinking alcoholic beverages recoded from 0–7 days to “None,” “1/2 days,” and “3/7 days” for analysis. Cancer screening was assessed with the questions “For females: How long ago did you have your most recent Pap test to check for cervical cancer?” with responses dichotomized to within 5 years and over 5 years or never, and analysis for this variable was limited to female respondents under 65, and “How interested are you in having a cancer screening test in the next year?” with the options not at all, a little, somewhat, very, or N/A/I am up to date with screening divided into “not at all/a little,” “somewhat/very” and “I am up to date” for analysis.

Sociodemographic factors were used as covariates in our analysis: gender (male/female), race (White/Black/Hispanic/Other (consisting of Native American or Alaska Native, Asian, Native Hawaiian or Pacific Islander, and Non-Hispanic multiple races mentioned)), age (18–34/35–49/50–64/66–74/75+), education (high school diploma or less/some college/college graduate), income (less than $35,000/$35,000-$75,000/more than $75,000), and cancer history (Had cancer/Never had cancer).

### Statistical analysis

Weighted descriptive statistics for each variable were analyzed. Multivariable ordinal regression models were used to explore the association between the dependent variables and independent and sociodemographic variables. Models containing independent variables controlled for the sociodemographic variables (i.e., gender, race, age, education, income, cancer history). We used complete case analysis to account for missing values. Results were considered significant for *p* values less than 0.05. All analyses were conducted using Stata version 17 (2021, StataCorp LLC, College Station, Texas).

## Results

The majority of the sample was female, Non-Hispanic White, and never had cancer (Table 1).

**Table 1:** Sample demographics

	Total (*n* = 6252) *n* (weighted %)
Gender	
Male	2307 (49.2)
Female	3535 (50.8)
Age	
18–34	939 (25.9)
35–49	1240 (25.3)
50–64	1772 (27.3)
65–74	1356 (13.0)
75 or older	847 (8.6)
Race/Ethnicity	
Non-Hispanic White	3203 (61.2)
Non-Hispanic Black	889 (11.0)
Hispanic	1001 (17.1)
Non-Hispanic Other	472 (10.8)
Education	
High school graduate and below	1455 (28.5)
Some college	1672 (38.9)
College graduate	2721 (32.6)
Income	
Less than $35,000	1688 (25.5)
$35,000–$75,000	1669 (30.1)
Greater than $75,000	2163 (44.4)
Ever had cancer	
No	4982 (89.9)
Yes	900 (10.1)

**Fig. 1:**
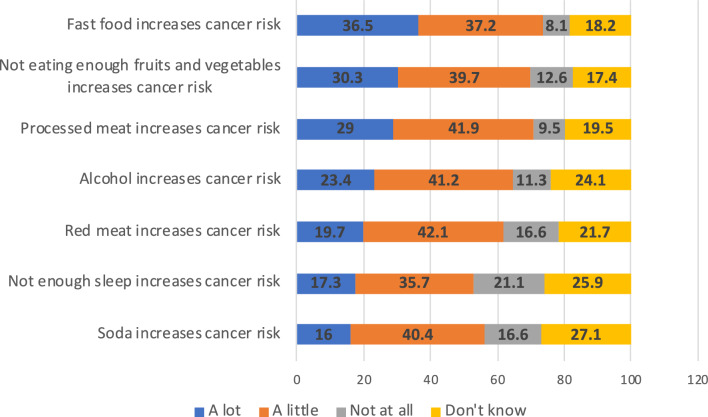
Cancer risk beliefs by frequency

When asked which behaviors increase cancer risk, the behavior that the highest percentage of respondents said increased cancer risk “A lot” was eating fast food (36.5%) (Fig. 1). The factor that the second highest percentage of respondents believed increased cancer risk “A lot” was not eating enough fruits and vegetables (30.3%). Drinking soda (16.0%) and not getting enough sleep (17.3%) were the factors that respondents were least likely to say increased cancer risk a lot. Behaviors with the highest percentage of respondents saying they did not increase cancer risk at all were not getting enough sleep (21.1%), eating red meat (16.6%), and drinking soda (16.6%). Drinking soda (27.1%) and not enough sleep (25.9%) had the highest percentage of respondents who answered “Don’t know.”

The logistic regression models showed higher odds among Hispanic respondents compared to Non-Hispanic White survey respondents of believing that soda increases cancer risk a lot (OR (95% CI) 1.69 (1.20–2.38)) (Table 2). Female respondents and Hispanic respondents had higher odds of believing drinking alcohol increases cancer risk a lot (all *p* < 0.05). There were higher odds of believing processed meat and believing red meat increases cancer risk a lot among female respondents, those aged 65–74, Black, and Hispanic respondents, while college graduates and Non-Hispanic Other race respondents only had higher odds of believing processed meat increases cancer risk (all *p* < 0.05). Female, Hispanic, and Non-Hispanic Other race respondents had higher odds of believing fast food increases cancer risk a lot (all *p* < 0.05). Those 65–74 and 75 or older, non-Hispanic Black, Hispanic, and college graduate respondents had higher odds of believing not eating enough fruits and vegetables increases cancer risk a lot (all *p* < 0.05). Those who had higher odds of believing not enough sleep increases cancer risk were respondents with a bachelors degree or more compared to those with a high school education or less (OR (95% CI) 1.57 (1.15–2.15)).

**Table 2:** Cancer risk beliefs by demographics

	Adjusted ordinal OR (95% CI) for drinking soda increasing cancer risk	Adjusted ordinal OR (95% CI) for drinking alcohol increasing cancer risk	Adjusted ordinal OR (95% CI) for processed meat increasing cancer risk	Adjusted ordinal OR (95% CI) for red meat increasing cancer risk	Adjusted ordinal OR (95% CI) for fast food increasing cancer risk	Adjusted ordinal OR (95% CI) for not enough fruits and vegetables increasing cancer risk	Adjusted ordinal OR (95% CI) for not enough sleep increasing cancer risk
Gender							
Male	Ref	Ref	Ref	Ref	Ref	Ref	Ref
Female	1.02 (0.84–1.25)	**1.52 (1.21–1.92)**	**1.30 (1.05–1.61)**	**1.27 (1.03–1.57**)	**1.44 (1.17–1.78)**	1.00 (0.88–1.30)	1.00 (0.80–1.25)
Age							
18–34	Ref	Ref	Ref	Ref	Ref	Ref	Ref
35–49	1.01 (0.68–1.52)	0.80 (0.52–1.24)	1.18 (0.84–1.65)	1.03 (0.74–1.42)	0.78 (0.58–1.06)	1.25 (0.90–1.75)	0.91 (0.66–1.27)
50–64	1.01 (0.71–1.44)	0.80 (0.52–1.25)	1.30 (0.90–1.87)	1.28 (0.88–1.84)	1.10 (0.79–1.52)	1.23 (0.91–1.67)	0.89 (0.66–1.20)
65–74	0.90 (0.61–1.32)	0.92 (0.59–1.44)	**1.81 (1.25–2.61)**	**1.85 (1.26–2.70)**	1.09 (0.79–1.51)	**1.97 (1.35–2.87)**	0.94 (0.66–1.35)
75 or older	0.74 (0.47–1.17)	1.32 (0.80–2.17)	1.26 (0.85–1.85)	1.51 (0.95–2.39)	0.93 (0.68–1.27)	**1.72 (1.19–2.48)**	1.09 (0.72–1.66)
Race/ethnicity							
Non-Hispanic White	Ref	Ref	Ref	Ref	Ref	Ref	Ref
Non-Hispanic Black	1.20 (0.75–1.92)	1.51 (1.00–2.29)	**1.94 (1.30–2.91)**	**2.41 (1.68–3.47)**	1.14 (0.82–1.57)	**1.62 (1.11–2.37**)	1.05 (0.74–1.49)
Hispanic	**1.69 (1.20–2.38)**	**1.70 (1.15–2.53)**	**2.29 (1.60–3.28)**	**2.37 (1.72–3.27)**	**1.94 (1.44–2.61)**	**1.50 (1.04–2.16)**	1.34 (0.96–1.89)
Non-Hispanic other	0.94 (0.53–1.65)	1.29 (0.72–2.30)	**1.78 (1.19–2.66)**	1.45 (0.90–2.33)	**1.64 (1.07–2.52)**	1.06 (0.77–1.44)	1.39 (0.92–2.09)
Education							
High school graduate and below	Ref	Ref	Ref	Ref	Ref	Ref	Ref
Some college	0.89 (0.62–1.29)	0.90 (0.65–1.25)	1.17 (0.85–1.62)	0.82 (0.60–1.14)	1.08 (0.80–1.44)	1.35 (0.98–1.85)	1.30 (0.94–1.81)
College graduate	1.06 (0.73–1.54)	1.18 (0.82–1.68)	**1.53 (1.11–2.10)**	1.20 (0.88–1.63)	1.29 (0.98–1.69)	**1.50 (1.09–2.07)**	**1.57 (1.15–2.15)**
Income							
Less than $35,000	Ref	Ref	Ref	Ref	Ref	Ref	Ref
35,000–$75,000	0.89 (0.69–1.44)	0.78 (0.55–1.11)	1.10 (0.84–1.45)	0.90 (0.68–1.19)	0.95 (0.73–1.22)	0.91 (0.70–1.18)	1.01 (0.76–1.35)
Greater than $75,000	1.06 (0.88–1.80)	0.90 (0.67–1.22)	1.33 (0.94–1.88)	1.11 (0.84–1.47)	1.18 (0.88–1.57)	0.92 (0.70–1.20)	0.92 (0.66–1.29)
Cancer history							
Never had cancer	Ref	Ref	Ref	Ref	Ref	Ref	Ref
Cancer survivor	1.32 (0.97–1.61)	1.10 (0.87–1.39)	1.06 (0.82–1.37)	1.17 (0.92–1.48)	1.00 (0.76–1.30)	1.28 (0.99–1.65)	1.05 (0.83–1.33)

Bold indicates significance at the *p* < 0.05 level

Controlling for demographic variables, there were significantly higher odds of believing drinking soda (OR (95% CI) 1.49 (1.16–1.91)), drinking alcohol (OR (95% CI) 1.82 (1.28–2.59)), eating processed meat (OR (95% CI) 1.84 (1.11–3.04)), eating red meat (OR (95% CI) 1.67 (1.24–2.24)), and not eating enough fruits and vegetables (OR (95% CI) 1.39 (1.01–1.92)) increases cancer risk among those who feel “A lot” or “Some” progress has been made in preventing cancer (Table 3). Those who think “A lot” or “Some” progress has been made in curing cancer had higher odds of believing drinking alcohol, not eating enough fruits and vegetables, and not enough sleep increases cancer risk (all *p* < 0.05). Compared to those who drink alcohol 0 days per week, those who drink 1–2 days (OR (95% CI) 1.63 (1.10–2.40)) or 3–7 days (OR (95% CI) 1.54 (1.10–2.16)) had higher odds of believing drinking soda increases cancer risk, and those who those who drink alcohol 1–2 days (OR (95% CI) 1.62 (1.05–2.49)) had higher odds of believing alcohol increased cancer risk. There were no significant relationships between hours of sleep per night or noticing calorie information on menus and cancer risk beliefs. Female respondents under 65 who had Pap tests within the past 5 years had higher odds of believing insufficient sleep increased cancer risk (OR (95% CI) 1.78 (1.07–2.98)). Respondents who were somewhat or very interested in getting a cancer screening in the next year had higher odds of believing processed meat, fast food, not eating enough fruits and vegetables, and not getting enough sleep increases cancer risk than those who were not at all or a little interested in getting screened for cancer. Those who agree there are too many cancer prevention recommendations did not have any higher odds of any cancer risk beliefs. Believing there is not much to do to lower chances of getting cancer was associated with lower odds of believing drinking soda, processed meat, red meat, and fast food increases cancer risk (all *p* < 0.05). Those who agreed that “When I think of cancer, I think of death” did not have any significant differences in cancer risk beliefs than those who disagreed. Those who reported thinking it seems like everything causes cancer had higher odds of believing drinking soda increases cancer risk (OR (95% CI) 1.53 (1.17–2.01)).

**Table 3:** Cancer risk beliefs by behavior and cancer fatalism

	Adjusted ordinal OR (95% CI) for drinking soda increasing cancer risk	Adjusted ordinal OR (95% CI) for drinking alcohol increasing cancer risk	Adjusted ordinal OR (95% CI) for processed meat increasing cancer risk	Adjusted ordinal OR (95% CI) for red meat increasing cancer risk	Adjusted ordinal OR (95% CI) for fast food increasing cancer risk	Adjusted ordinal OR (95% CI) for not enough fruits and vegetables increasing cancer risk	Adjusted ordinal OR (95% CI) for not enough sleep increasing cancer risk
Progress has been made in preventing cancer							
A little/almost none	Ref	Ref	Ref	Ref	Ref	Ref	Ref
A lot/some	**1.49 (1.16–1.91)**	**1.82 (1.28–2.59)**	**1.84 (1.11–3.04)**	**1.67 (1.24–2.24)**	1.30 (0.84–2.02)	**1.39 (1.01–1.92)**	1.16 (0.88–1.54)
Progress has been made in curing cancer							
A little/almost none	Ref	Ref	Ref	Ref	Ref	Ref	Ref
A lot/some	1.25 (0.87–1.79)	**1.58 (1.10–2.26)**	1.48 (0.97–2.28)	**1.56 (1.11–2.19)**	1.13 (0.71–1.80)	**1.52 (1.14–2.05)**	**1.34 (1.06–1.69)**
Days per week drinking alcohol							
0 days	Ref	Ref	Ref	Ref	Ref	Ref	Ref
1–2 days	**1.63 (1.10–2.40)**	**1.62 (1.05–2.49)**	1.34 (0.79–2.29)	1.22 (0.89–1.68)	1.39 (0.75–2.60)	1.06 (0.67–1.67)	1.08 (0.75–1.55)
3–7 days	**1.54 (1.10–2.16)**	1.16 (0.63–2.13)	1.38 (0.68–2.82)	1.42 (0.95–2.13)	1.13 (0.60–2.14)	0.68 (0.43–1.07)	1.19 (0.83–1.71)
Hours of sleep per night							
5 or less	Ref	Ref	Ref	Ref	Ref	Ref	Ref
6–7	1.04 (0.66–1.64)	0.82 (0.46–1.45)	1.11 (0.65–1.91)	1.08 (0.74–1.58)	0.79 (0.47–1.33)	1.12 (0.73–1.72)	1.20 (0.83–1.75)
8 or more	0.75 (0.47–1.19)	0.67 (0.36–1.23)	0.85 (0.49–1.48)	1.19 (0.77–1.82)	0.81 (0.44–1.47)	0.94 (0.60–1.47)	1.09 (0.75–1.57)
Notice calorie info on menus							
No	Ref	Ref	Ref	Ref	Ref	Ref	Ref
Yes	1.03 (0.76–1.39)	1.35 (0.96–1.90)	1.35 (0.91–2.01)	1.00 (0.75–1.32)	0.98 (0.65–1.49)	0.98 (0.68–1.40)	0.97 (0.75–1.26)
Pap test within past 5 years (among female respondents 18–64)							
Not in past 5 years/never	Ref	Ref	Ref	Ref	Ref	Ref	Ref
Test in past 5 years	0.81 (0.52–1.28)	1.33 (0.63–2.82)	1.30 (0.60–2.83)	1.16 (0.59–2.30)	1.27 (0.61–2.63)	1.38 (0.75–2.55)	**1.78 (1.07–2.98)**
Interested in getting a cancer screening within the next year							
Not at all/a little	Ref	Ref	Ref	Ref	Ref	Ref	Ref
Somewhat/very interested	1.07 (0.73–1.57)	1.43 (0.91–2.26)	**1.49 (1.04–2.15)**	1.12 (0.76–1.67)	**1.51 (1.02–2.24)**	**1.48 (1.03–2.13)**	**1.46 (1.09–1.95)**
I am up to date with screening	0.82 (0.51–1.31)	0.72 (0.42–1.26)	0.97 (0.43–2.18)	0.95 (0.58–1.55)	0.98 (0.51–1.88)	1.10 (0.72–1.66)	1.07 (0.75–1.54)
So many recommendations it’s hard to know which to follow							
Strongly/domewhat disagree	Ref	Ref	Ref	Ref	Ref	Ref	Ref
Strongly/somewhat agree	0.99 (0.74–1.32)	1.16 (0.75–1.79)	1.10 (0.74–1.65)	1.00 (0.74–1.35)	1.46 (0.95–2.25)	0.84 (0.59–1.19)	1.01 (0.75–1.35)
There’s not much you can do to lower chances of getting cancer							
Strongly/somewhat disagree	Ref	Ref	Ref	Ref	Ref	Ref	Ref
Strongly/somewhat agree	**0.59 (0.43–0.80)**	0.67 (0.43–1.03)	**0.47 (0.31–0.73)**	**0.55 (0.39–0.78)**	**0.53 (0.35–0.82)**	0.68 (0.46–1.00)	0.78 (0.56–1.08)
When I think about cancer, I think about death							
Strongly/somewhat disagree	Ref	Ref	Ref	Ref	Ref	Ref	Ref
Strongly/somewhat agree	1.15 (0.89–1.47)	1.42 (0.97–2.09)	1.16 (0.88–1.54)	1.35 (0.99–1.85)	1.35 (0.98–1.86)	0.98 (0.76–1.27)	0.90 (0.71–1.13)
It seems like everything causes cancer							
Strongly/somewhat disagree	Ref	Ref	Ref	Ref	Ref	Ref	Ref
Strongly/somewhat agree	**1.53 (1.17–2.01)**	1.31 (0.83–2.07)	1.45 (0.91–2.33)	1.15 (0.82–1.61)	1.32 (0.93–1.87)	1.36 (0.94–1.98)	1.16 (0.88–1.51)

Bold indicates significance at the *p* < 0.05 level

## Discussion

There were demographic differences in cancer risk beliefs, with female, Black, Hispanic, Other race, those aged 65–74, and college graduates having higher odds of believing multiple behaviors increase cancer risk. Those who believe progress has been made in cancer prevention and curing cancer also have higher odds of believing behaviors increase cancer risk. The behaviors people most frequently thought increased cancer risk “A lot” were eating fast food and not eating enough fruits and vegetables, while drinking soda and inadequate sleep were more often thought to “Not at all” increase risk or elicit a “Don’t know” response. Those who drink alcohol 1–2 days per week had higher odds of believing alcohol increases risk, but those who get more hours of sleep did not have higher odds of believing lack of sleep was a cancer risk and those who notice calorie information on menus did not have different odds of believing soda, fast food, processed meat, red meat, or insufficient fruits and vegetables increases cancer risk. Those who had Pap tests within the previous 5 years and those who were interested in receiving cancer screenings had higher odds of believing certain behaviors increased risk.

People who were races other than White were more likely to believe that poor diet and alcohol increase cancer risk. Past research shows cancer prevention behaviors were less frequent among racial and ethnic minority populations, so it may not be awareness, but other factors leading to these disparities [5–7]. Disparities including inequitable access to fresh fruits and vegetables, fewer grocery stores, and increased access to fast food are more likely to affect non-White, lower socioeconomic status, urban, and/or rural populations [5, 7, 23]. Cancer prevention recommendations often encourage individual behavior change, which may be harder in areas with more barriers to healthy diet, exercise, and regular health screenings [24]. In this study, the demographics less likely to believe risk behaviors affected cancer risk were non-Hispanic White and male respondents. Educational campaigns could be tailored to these demographics to enhance knowledge about cancer risk factors among these groups that have lower perceived cancer risks. It is necessary to develop and implement multi-level interventions based on contextual factors, policy changes, and community investment programs, as well as to incorporate social support to increase educational engagement and the likelihood of long-term behavioral changes [24, 25].

In this study, fatalism was not consistently linked to beliefs about behaviors affecting cancer risk, with only one behavior thought to increase cancer, soda consumption, linked to the fatalistic belief that it seems like everything causes cancer. Believing there is not much you can do to lower risk of cancer was linked to lower odds of believing soda, processed meat, red meat, and fast food increase cancer risk, indicating these respondents are less likely to link behaviors to cancer. There is some evidence in prior research that those with fatalistic cancer beliefs are more likely to have risky behaviors like smoking or drinking alcohol, low fruit and vegetable consumption, and low exercise [15, 16, 26, 27]. Fatalistic beliefs may lead people to believe their behavior does not matter, or that if cancer can be affected by behavior it is inevitable if there are barriers to behavior change [18, 28]. Awareness alone may be insufficient to change how people behave, and cancer prevention efforts may need to help encourage behavior change through training and tailored recommendations [29]. For example, while people are aware that vegetables are healthy, they may need information about where to acquire them, interventions to help them learn how to prepare them, culturally and skill-concordant recipes, and modeling of positive behaviors from others [29, 30].

There were not many associations between believing behaviors caused cancer and the actual alcohol, sleep, and calorie information seeking behaviors of HINTs respondents, while interest in cancer screening had several associations with perceiving poor diet and little sleep as cancer risks. There may be a disconnect between knowledge of the risk and actually confronting the risk. Prior research shows that self-efficacy may have an effect on translating risk information to behavior, but those with low-self-efficacy may actually avoid cancer risk information and information regarding threats that they consider uncontrollable [31]. Our findings that interest in cancer screening is associated with believing in more behavioral risks corroborate that there is less information avoidance in those who feel more control over their cancer risk. Beliefs about the cause and controllability of cancer likely influence risk reduction behaviors. In prior research, causal beliefs of cancer survivors have been found to differ compared to those with no personal history of cancer and those with only family history, with cancer surivors underestimating the role of behavioral factors such as physical activity and poor diet, and overestimating the role of environmental pollutants and stress [32]. Similarly, in our study, cancer survivors were not significantly more likely to believe any personal behaviors increase cancer risk. Those with a personal history of cancer do not want to feel at fault, while at the same time those with a family history or no cancer may want to feel control over their future cancer risk [33–35]. Health educators need to ensure cancer prevention messages are supportive of behavior change without alienating cancer survivors and contributing to fatalistic beliefs [28, 33–35].

### Limitations

Although HINTS provides a large, nationally representative sample, this study only used one iteration of HINTS, as the questions regarding which behaviors affect cancer risk were only asked in the 2022 survey. Additional data from future iterations may provide sufficient data to show demographic trends and trends over time. Moreover, there were few personal nutrition questions on the 2022 HINTS survey, so consumption of fruits and vegetables, fast food, soda, and red or processed meat could not be assessed, only whether people pay attention to calorie information and the risk opinions regarding these behaviors. In addition, this survey data is subject to social desirability bias and recall bias, especially for behavior and beliefs about behavior, as people tend to report their behavior at their healthiest times. Finally, while race and ethnicity are included, there are many cultural and social factors that may influence beliefs that cannot be easily ascertained using only survey data. Additionally, we were unable to ascertain some racial differences because some races had fewer than 300 survey respondents and were combined into the Other category, including Native American, Asian, Native Hawaiian or Pacific Islander, and Non-Hispanic multiple race respondents.

## Conclusion

The majority of people believe nutrition, alcohol, and sleep have some effect on cancer risk but women, older adults, and races other than white are more likely to believe many of these behaviors affect risk. Those who believe these behaviors affect risk may not actually perform more cancer prevention behaviors, as there may be barriers to changing behavior to prevent cancer. Cancer prevention information should help inform which behaviors affect risk but also focus on increasing self-efficacy and helping people practically make changes. It is important to develop behavior change interventions to support these changes, while being careful not to overwhelm people and potentially increase fatalistic beliefs like “it seems like everything causes cancer.” By avoiding information overload, having tailored information, providing positive messaging, and targeting specific behaviors, such interventions may hold promise supporting cancer prevention behaviors across populations.

## Data Availability

All data used in this study was from the publicly available Health Information National Trends Survey. Data and information about this survey can be found at https://hints.cancer.gov.
